# Coping with migration-related stressors - a qualitative study of Nepali male labour migrants

**DOI:** 10.1186/s12889-021-11192-y

**Published:** 2021-06-12

**Authors:** Joelle Mak, Cathy Zimmerman, Bayard Roberts

**Affiliations:** London School of Hygiene & Tropical Medicine, Faculty of Public Health & Policy, 15 – 17 Tavistock Place, London, WC1H 9SH UK

**Keywords:** Migrant workers, Nepal, Coping, Stressors, Exploitation

## Abstract

**Background:**

International labour migration has become a crucial livelihood strategy, especially in countries where decently paid employment opportunities are limited. Such opportunities come with many potential benefits but also many stressors that challenge migrants’ coping skills, especially when they are in a foreign environment away from their normal support network. This paper explores how labour migrants coped with migration-related stressors using a sample of male Nepali migrants.

**Methods:**

Qualitative life histories were conducted in Kathmandu among returnee male migrants. Coping responses were categorised based Skinner and Zimmer-Gembeck’s coping typologies. The interview scripts were transcribed in Nepali and translated into English for analysis. Each interview script was open coded and then categorised according to the 12 core coping families. Data were analysed thematically to explore relationships across and within coping and stressors.

**Results:**

Forty-two men were interviewed who mainly worked in low- and semi-skilled jobs in Malaysia, and the Gulf States. The coping strategies most commonly used belonged to the families of problem-solving, support-seeking, negotiation and helplessness. Men used these either individually or collectively with other migrants. Those who sought assistance from authorities or civil society organisations did not always receive the help needed and there were mixed messages as to when and what types of assistance were available. Some stressors involved multiple coping strategies simultaneously, others described changing strategies following unsuccessful earlier attempts. The coping families of helplessness and social isolation reflected migrants’ limited power in challenging certain stressors. The choice of coping strategies was also moderated by factors such as outstanding loans, language difficulties, or not wanting to cause their family distress. Some coping strategies used led to  new stressors.

**Conclusions:**

Migrants need greater clarifications on their rights with respect to contract discrepancies, the types of support available, how and from whom to access them once in destination. Improvements to the support mechanisms migrants can access as well as strengthening migrant-led initiatives in destination countries to support labour migrants' in managing stressors are needed. These may contribute to reducing the experiences and impact of such stressors, which may ultimately lead to more successful migration outcomes. As labour migration from Nepal is likely to continue, government and CSOs need to ensure migrants have the support they need to cope with the challenges they may encountered along the way.

**Supplementary Information:**

The online version contains supplementary material available at 10.1186/s12889-021-11192-y.

## Background

International labour migration has become an important livelihood strategy for individuals in low- and middle-income countries (LMIC) where employment opportunities, particularly for low- and semi-skilled work, are limited or poorly paid. Through labour migration, individuals have the potential to better provide for their families, sometimes earning wages well above what is possible in their own country. These opportunities make the high upfront financial costs of migration a worthwhile investment. Many are subsequently able to acquire land, pay for children’s education and access healthcare [1–3].

At the same time, many labour migrants encounter financial, social and emotional challenges from pre-departure, at destination to their eventual return home. These include financing the migration followed by potential issues in destination such as contract breaches, poor living and working conditions, restrictions of movement, and document confiscation [4–8]. Labour migration is often circular, where workers migrate repeatedly between their home country and work destinations [9]. In Nepal, international labour migration is a common employment strategy, with males in low-skilled work making up the vast majority of those migrating. Although labour migration has become more prevalent among Nepali women, it is still an overwhelmingly male activity. Men received approximately 95% of the official labour permits issued annually and this figure has remained consistent over the past 10 years [10]. However, it is widely acknowledged that many women may migrate through irregular channels without applying for official permits and the various bans restricting women’s labour migration movements have impacted on low official figures [10, 11].

Studies have found that labour migration among men, including Nepali men, can be seen as a rite of passage as many migrate for the first time in late adolescence and return as older adults. For many men, international migration exposes them to discrimination, based on racial or ethnic lines or on their status as labour migrants [12, 13]. In some cases, migrants may be educated or from a higher status group in their own country, which makes the experience of being treated as lower status even more testing. In other cases, the reverse may be true in that men are able to escape discrimination, for example based on caste, by migrating to a country where they are seen and treated like any other Nepali labour migrant, as highlighted in an ethnographic study of the Kamaiya, a group traditionally linked to bonded labour in Nepal [14]. Men may also be seen by society and indeed, by themselves, as less vulnerable to trafficking, due to the prevailing norms of hegemonic masculinities as well as the traditional narrative between trafficking and sexual exploitation. Research among trafficked men in Eurasia and South-Eastern Europe found that men attached significant stigma to being ‘trafficking victims’, believing that only ‘stupid’ men could be trafficked and unable to get out of the situation themselves [15]. Such expectations and beliefs may result in men’s reluctance to accept assistance or support, if doing so labels them as having been trafficked.

There is a large and growing body of literature that has described the numerous abuses associated with exploitation and human trafficking, as well as the adverse outcomes related to individual’s health. Systematic reviews have noted poor physical, mental, sexual health among survivors of human trafficking [16] and labour migrants [17]. Ottisova et al’s updated systematic review noted that in their original 2012 review, there was little evidence focussing on men who had been trafficked or those trafficked for labour exploitation but research has expanded since. Additionally, migrants often work in physically demanding jobs and may be disproportionately exposed to increased occupational risks. Studies comparing migrant and host populations have found that migrants suffer from more work-related strains and injuries [18, 19]. Further, an increasing number of work-related disabilities and fatalities among Nepali migrants working abroad, have also been highlighted [5, 17, 20]. According to Nepal’s Foreign Employment Promotion Board (FEPB), between 2008/9 and 2018/19, nearly 7500 Nepali labour migrants died while working abroad (7296 men and 171 women). The main officially recorded causes of death included: 22% to ‘natural causes’; 18% cardiac arrest; 14% road accidents; 12% suicide; 9% workplace accidents; 8% heart attack; and 18% other causes [10] although post mortems were rarely carried out [21].

The WHO defines health in a holistic way, beyond a simple absence of disease [22]. However, this definition does not acknowledge the ability for humans to cope with changing circumstances [23]. Coping has an important role in the attainment of good health, particularly in situations where full recovery from ill-health may not be a realistic outcome [23]. Wellbeing, therefore, should be seen as an ‘equilibrium’ between an individual’s resources, such as coping, to face psychological, social and physical challenges [24]. Accordingly, for individuals in situations of exploitation, increasing their coping resources to manage various stressors can lead to improvements in their overall wellbeing; whereas poor coping can result in greater ill-health, including mental ill-health.

Although many of the stressors faced by migrants have been documented, how they cope under such circumstances, away from their normal social support network, has been less well-researched. This paper aims to fill that gap by exploring coping strategies migrants used to manage stressors encountered throughout the migration cycle, and how they varied for different types of stressors and circumstances.

## Defining and categorising coping strategies

Coping strategies are the ways individuals respond to stress and stressful situations. Coping has been conceptualised as individual personality traits that are relatively stable characteristics; or as a process that changes over time, influenced by the context of the stressors [25, 26]. Strategies themselves are sometimes defined and measured by their function, most commonly problem-focused or emotion-focused with the former aimed at confronting the source of the stressor and the latter at changing one’s reactions to them [25]; or by active versus passive or avoidance coping [27]. However, it is recognised that many coping strategies serve multiple functions simultaneously and are not sufficiently reflected when categorised by such binaries [28].

The different ways in which coping has been conceptualised have led to the development of different measurement tools and labelling systems that have made comparisons between studies difficult [29, 30]. Skinner and colleagues reviewed coping measures from different empirical studies and identified 100 coping categories. These were then synthesised into 12 core ‘coping families’ [30]. The twelve families are grouped into three sets of processes: coordinating actions, resources and options. The coping families are: problem-solving, information-seeking, helplessness, escape, self-comforting, support-seeking, delegation, social isolation, accommodation, negotiation, submission and opposition. These are described below and summarised in Table 1.

**Table 1:** Description of core coping families (adapted from Skinner & Zimmer-Gembeck [29])

Purpose	Coping families	Description
Coordinate action	Problem-solving	active attempts to achieve desired outcomes through: strategizing; planning; analysing; preventing; repairing
	Information-seeking	active attempts to gather relevant information (causes, meaning, consequences) of the stressor(s) through: seeking advice or help; observing or consulting others or relevant materials
	Helplessness	give up control of a situation without attempts to improve situation through being passive; resigning
	Escape	remove oneself from stressor through: denial; physically leaving; cognitively avoiding
Coordinate resources	Self-comforting	engage in active self-care through: relaxing; controlling one’s own emotions constructively; encouraging oneself
	Support-seeking	seek support from other individuals or religion through: reaching out to others; seeking comfort; imagining the response of others; praying
	Delegation	heavy reliance on others’ support and focuses negatively on the stressor through: complaining; whining; maladaptive help-seeking
	Social isolation	withdraw from others physically or emotionally to prevent others knowing about stressor or their effects, often due to sadness or shame
Coordinate options	Accommodation	adjust preference to available options through: positive cognitive restructuring; distraction
	Negotiation	active attempts to compromise, focussing on defending one’s goals through: bargaining; reducing demands; priority setting; deal-making
	Submission	surrender to stressor through: rumination; negative thinking; catastrophizing; self-blame
	Opposition	attack source of stressor combined with anger or hostility through: using aggression; blaming others; taking revenge; being defiant

Problem-solving involves strategising, planning and taking direct action at the stressors. Information-seeking attempts to understand the causes of the stressors, and is generally used to cope with stressors that are unfamiliar. Helplessness is to give up control of stressful situations and give in to feelings of confusion, exhaustion, and doubt. Escape coping avoids stressors through denial, cognitively or behaviourally, and are usually based on fear. Self-comforting strategies focuses on self-care and relaxation to prevent the stressors from taking hold. Support-seeking coping is to turn to others for emotional, instrumental or spiritual support. Delegation tends to be used when individuals concentrate on the negative features of the stressors and rely on self-pity or complain. Social isolation can be physical or emotional, and deliberately prevents others from knowing about the stressful situation, due to despondency or embarrassment. Accommodation is used to regulate one’s emotions or assessment of the situation to a more positive one, or through distractions by taking part in pleasurable activities. Negotiation seeks a compromise through bargaining or persuasion while protecting one’s interests. Submission strategies resign to stressors by engaging in rumination, catastrophising or self-blame. Finally, opposition involves aggressively confronting the stressor and its source, in defiance or retaliation, by venting or blaming others.

## Methods

This study used life history, with an emphasis on participant’s migration experiences, to examine men’s lives. According to Singer, ‘as individuals narrate their life story, they reveal not just the circumstances of their life, but the backdrop of values and commitments that make that life meaningful to them’ ([31], p. 449). Further, in narrating their life story individuals share information related to the wider cultural and structural influences in decision-making throughout one’s lifespan [31]. Life history also enables conversations to start on a more neutral ground which may helped to develop rapport.

Semi-structured topic guides were developed that followed a chronological approach, beginning by exploring participants’ childhood, upbringing, family, community (village) life and schooling, to the early phases of their working life, how they came to consider migration, their family’s feelings, how men navigated the migration process, as well as their experiences at destination and their return along with repeated migrations (Supplementary file). Other important life milestones such as marriage, fatherhood, and caring with aging parents or other pressures at home were also explored. The guide was revised as issues emerged from earlier interviews.

This study focused on male returnee migrants as the vast majority of labour migrants from Nepal, according to official records, are males. Additionally, an earlier exploration of returnee migrants using a household census identified very few women [32]. Therefore, in this study we focused on men’s experiences.

Semi-structured interviews were conducted with 42 adult male Nepali migrants over the age of 18, who had worked outside of Nepal. We focused on returnee migrants, first because of the difficulty of reaching the wide range of countries to which Nepalis migrate, most commonly Malaysia and the Gulf States. Additionally, once migrants have returned home, they are generally much freer to disclose their experiences than while in foreign contexts where they might be more restricted or fear harm from speaking to researchers. Furthermore, it is more difficult to gain less biased samples in the destination countries, because the most exploited individuals are likely to be much less accessible.

Participant recruitment was conducted at hotels near Kathmandu’s airport and main bus stations. Nepal has only one international airport. On their return to Nepal, many international returnee migrants stay overnight near the main bus stations to connect with transport back to their villages the next day. It is, therefore, relatively easy to get in touch with them there. Four areas near the airport and main bus stations were identified: Sinamangal, Gongabu, Sundhara, and Gaushala/Battisputali. Sampling was conducted through the lodges in these areas, first by visiting the area to identify lodges. Next, each lodge was visited to introduce the study and to seek permission to recruit participants at their premises. Owners were also asked if they knew of other lodges nearby where returnee migrants might stay. Additionally, local migrant organisations were contacted for referrals of other potential participants to capture a broad range of experiences. Most interviews took place in the men’s hotel rooms or at the interpreter’s home.

The fieldwork took place between February and May 2016. Interviews were conducted in English, with the assistance of an interpreter, and were audio-recorded. Data collection concluded when saturation was reached, with the final interviews not reflecting sufficiently different narratives. The interviews took on average two hours, ranging from 35 min to 2.5 h. Notes were also taken during the interviews and at the end of each fieldwork day a summary was written documenting that day’s work, including the process, impressions and reflections.

### Ethics

Ethical approval was obtained from the London School of Hygiene & Tropical Medicine (ref: 1040) and the Nepal Health Research Council (ref: 7021). Participants were given study information verbally and in writing, and provided written consent before interviews began. At the end of the interview participants were given 500 NPR (approximately $5.00 USD) as a token of appreciation.

### Data analysis

The interview scripts were translated into English for analysis. Each interview script was first read and re-read in full, along with the interview summaries and fieldnotes. Each interview was then open coded in NVivo 11.0, to develop an initial coding frame inductively, through ideas and concepts that emerged to produce a comprehensive representation of the data. Once all the interviews were open-coded, they were refined to focus on the ways in which participants described coping or managing with the various different challenges they encountered. The last stage involved categorising the codes according to the 12 core coping families [29, 30].

The coded data were then charted on a spreadsheet to create an overview of all the interviews, together with each participant’s socio-demographic information. Direct quotations and references to the original source of each interview were noted in the spreadsheet to preserve the overall context of the narrative, keeping the actual terminology used by participants to describe and define their experiences, as well as to facilitate subsequent retrieval of data for comparison between and within cases. Finally, thematic analysis was conducted to explore relationships across and within coping and stressors. Quotations are presented using pseudonyms.

## Results

### Study sample

The participants’ demographic and migration characteristics are presented in Table 2. The majority of the participants (60%) had multiple migration experiences. In their most recent migration, participants worked in a range of low- and semi-skilled occupations primarily in Qatar, Saudi Arabia and Malaysia.

**Table 2:** Demographic and migration characteristics of study sample

	n (%)
**Age-groups:**	
< 25	10 (23.8)
25–34	20 (47.6)
35–44	9 (21.4)
≥ 45	3 (7.1)
Full age range: 21–53	
**Caste/ethnicity:**	
Brahman/Chhetree	12 (28.6)
Janajati	15 (35.7)
Dalit	5 (11.9)
Other	10 (23.8)
**Area of origin:**	
Terai (lowland)	33 (78.6)
Hill	8 (19.0)
Mountain	1 (2.4)
**Highest level of education attended:**	
None/Informal/Primary	10 (23.8)
Secondary	24 (57.1)
Higher secondary/vocational/tertiary	8 (19.0)
**No. previous labour migrations:**	
Once	17 (40.5)
More than once	25 (59.5)
**Most recent migration destination:**	
Malaysia	12 (28.6)
Qatar	12 (28.6)
Saudi Arabia	11 (26.2)
United Arab Emirates	5 (11.9)
Kuwait	1 (2.4)
Afghanistan	1 (2.4)
**Most recent migration work:**	
General labourer/porter	16 (38.1)
Driver / carpenter / plumber / mason	10 (23.8)
Factory worker	6 (14.3)
Kitchen /food-related work	4 (9.5)
Security	4 (9.5)
Retail/office boy	2 (4.8)
Duration of most recent migration*:	
< 1 year	6 (14.3)
1 - < 3 years	18 (42.9)
3 - < 5 years	7 (16.7)
5 - < 10 years	8 (19.0)
≥ 10 years	3 (7.1)
**Next plans:**	
Return to the same job and employer	7 (16.7)
Re-migrate to different destination/job/company	22 (52.4)
Stay in Nepal	8 (19.0)
Undecided	5 (11.9)

### Problem-solving

The coping family of problem-solving involves analysing, strategizing and planning to achieve the desired outcome [29]. Men used this to manage a wide range of stressors beginning with the decision to migrate to cope with financial difficulties in Nepal, sometimes despite disagreement with their parents. Loans taken to cover basic living expenses were regularly described in men's narrative, reflecting their upbringing  in households that relied on subsistence farming.

Structural stressors included inconsistent electricity with frequent load shedding for half of the day and regular strikes in Nepal. One participant explained that this affected his ability to earn money as he was rarely able to work for more than 10–15 days per month. This led him to consider migration to earn a more regular income. He recognised the risk of indebtedness, as did many others, whose families had regularly taken out loans to meet household expenses. He opted instead to save enough money to cover the expenses in full before migrating.

One participant explained using migration to escape the Maoist political conflict after finding his details were published when he and some colleagues formed a union. He fled the country with the intention to return after six-months. However, subsequent financial problems at home meant he did not return for several years.

Once the decision to migrate was made, men needed to secure the finances, identify suitable jobs, recruitment agents or agencies and navigate through the process and regulations. Men reportedly obtained the funds to migrate through loans, using land or gold as collateral, or by selling assets, such as motorbike and livestock.

Once in a destination, nearly all participants experienced stressors related to their employment. These included contract discrepancies with worse terms and conditions, particularly in relation to wages, job and working conditions including hours and days of work and heavy workload. Initially, many tried to cope using problem-solving strategies such as refusing to work or to sign the new contracts. One participant ultimately decided the best solution was to return to Nepal, despite his outstanding loan.

…just paying the loan will not make that much of a difference. If these things happen there then I would rather go work in India. (Amar, age-group 25–34)

Many responded to wage-related problems including lower than agreed wages and delayed payments by reducing the frequency of sending remittances or by budgeting with their family in Nepal. Some men limited their own expenses by not going out, not returning to Nepal for vacation, or extending their contract to ensure their eventual flight home would be covered by their employer. Loans were key stressors for many; and an extended stay was a strategy used to cope with lower than expected wages, which resulted in a longer period required to repay loans, as described by one participant:

… if I go out I have to pay any expenses myself. And I was getting much less that what I was supposed to get. I had taken a loan to go there so I needed to save money to repay it. If I go out then there is expense on taxi, bus; so, I stayed in the room and watched TV. (Mahendra, age-group <25)

When faced with multiple stressors, or where one source of stress has implications for another, as in the above example, many participants coped by analysing, strategising and prioritising. Men prioritised repaying their loans rather than addressing the contract discrepancies, despite being aware of available resources, as one participant described:

…in orientation class [Nepal government mandated pre-departure training] they had said that in such cases the embassy can help us but I did not feel like going there. At first, I thought to clear my loan so after 14-15 months, I earned 400,000 NPR and I came back. (Sagar, age-group 25–34)

Participants in labour or factory work reported heavy and difficult workload; while others described having to work seven days a week throughout their entire contract period. Men made up excuses to have a day off, being careful not to do it too often to avoid conflict with supervisors.

I didn’t take sick leave. When they called me for work on Saturdays I would sometimes say I was sick if I was too lazy to go. I didn’t do that often, maybe only once a month. Calling in sick didn’t lose me any money as they never pay for work on Saturdays anyway. (Sagar, age-group 25–34)

Poor living conditions, including unhygienic, overcrowded or insecure facilities, were a source of stress for many. Men managed by forming groups to cook in turns, keeping their valuables, such as mobile phones and cash, on their person. Others described using the bathroom facilities at the workplace if they did not reach the head of the queue in their accommodation when the transport arrived to take them to the worksite.

Several participants who worked in factories reported occupational stressors including exposures and allergic reactions to dust and chemicals but were either provided with poor quality or no protective equipment at all. Participants coped by buying their own protective equipment.

When men tried to return to Nepal, many were not permitted to go, or could only go with a substantial financial penalty. For example, they may have to pay a fine for early termination of their contract, or have to leave a large deposit to guarantee their return. This was regardless of whether the leave request was due to illness or death in the family, termination of their contract, or on completion of their contract. As a result, many made up reasons to justify the need to return home to improve their chances of being granted leave. One participant described the wage differential that led to his desire to leave, and the consequences for doing so:

…the manpower [recruitment agency in Nepal] had said the salary will be 1,277 plus overtime and then after going there the salary was only 600… So, then I wrote an emergency application [making up reason to go home] and two month's salary was on hold. (Damodar, age-group 25–34)

Another participant reported being persistent with applying for leave despite having it dismissed by his manager on multiple occasions. It was only during a chance meeting with the owner that he realised that the owner had no objections to him leaving. Rather, it was the manager that did not want to authorise his leave.

I said I had family issues even though there were no such issues. I first went to the manager, he didn't listen and then I kept on asking him but nothing happened. Finally, I met with my boss by chance and told him that I wanted to go home and I got my passport back. … he called the manager and told him to arrange my cancellation documents, my immigration papers and I was allowed to leave after one week…. He [the boss] gave me 1,000 Dirham extra as well. (Mahendra, age-group <25)

Some reported that their employers would either not let them terminate their contract, or would delay their leave even when they had completed their contract. Some participants felt applying for vacation would be quicker and more likely to be approved. Several men used this strategy even when they had no intention of returning after the vacation. One participant decided early on to leave due to contract discrepancies and planned out how to convince his employer to grant him leave.

I had not finished the contract. Without finishing the contract, they wouldn’t let me come [to Nepal]. Dashain is big festival and I wanted to go at that time. I used to speak properly with them and do the work properly as well. Except for one or two days off I worked anytime they asked so they were very satisfied. That is why when I said I want to go home for Dashain holidays they trusted me and send me back. But after coming back I didn’t return. I came here taking holiday. (Sagar, age-group 25–34)

Men described deciding to stay until they could pay the fines demanded by their employer, or to repay their loans. Some employers withheld several months’ salary before approving migrants’ leave, as described by one participant:

I didn't come back after two years for holidays. I was able to but didn't because the company holds some salary back. If they [migrants] come on holiday they [company] is not sure if they will come back or not so for that they hold certain amount of money. (Janak, age-group 35–44)

In some cases, men also extended their contract after repaying their loans in order to return home with savings. Further, several men reported that their employers would only cover their airfare home if they signed a new contract. In these scenarios, participants signed the new contract, worked for a few months and then applied for leave, thus getting around paying for their flight, even though they had no intention of returning to complete their new contract.

### Helplessness

Helplessness strategies include passive coping where individuals give up control without attempting to improve their situation. Men used these strategies to manage financial difficulties, deceptions by recruitment agents or agencies, and contract discrepancies. For some, helplessness coping was only used after unsuccessful attempts with other strategies. Outstanding loans coupled with the fact that fees had already been paid often limited their options. Even those who had discovered before leaving Nepal that the recruitment agency had deceived them were unable to challenge them.

Contract discrepancy and associated financial stressors, such as having to pay fines if they did not agree to the new contract terms on arrival in the destination were sometimes addressed with helplessness strategies, as described by a participant who was asked to pay the equivalent of four months’ salary in order to leave:

If I had the money I would have given it to him very fast. If I had money, I would have left. ... But I couldn’t do anything. (Narendra, age-group 25–34)

In most cases, salary deductions were taken by the employers but migrants themselves did not always know who was actually keeping their wages. One participant only realised when his employer was closing down and he was being sent home early. He asked for the withheld wages that was meant to be paid on completion of his contract. He then discovered that it was the recruitment agency that had written the deductions in the contract to be paid to them.

They [employer] told us that we were made a fool. The agency had told the company to deduct the money. And HR said we even signed the document saying they should deduct it. It was right, we had signed it. The [agency] told us that when we return after two years, our 1,200 would be returned and we would get a bonus. So, we signed it. (Gyan, age-group 25–34)

Helplessness strategies were also used to cope with legal issues such as when confronted by the police. These were sometimes mediated by migrants’ limited language skills, unfamiliarity with the legal process, and lack of trust in the police force. Many participants had no faith that the police would help them under any circumstances:

We didn't know how to complain. We didn't know the language, and we knew about the police asking for money [bribes] as well so I didn't feel like going to them. (Amar, age-group 25–34)

Additionally, employers’ practice of confiscating passports or documents contributed to participants’ feelings of insecurity about going out. If they were stopped by the police they would not have any documents to proof their status. One participant who wanted to seek assistance at the embassy due to contract issues felt unable to do so without identification documents:

I talked to them [embassy and CSOs] but they also asked for ID. Police also asked for it. I did not go but my friends had gone so until I got my ID I just continued to work. (Bishnu, age-group <25)

Another, who was aware that contract discrepancies could arise had taken precautions by making photocopies of his contract signed in Nepal to send back to his father for safekeeping. However, he later discovered that they were insufficient to seek compensation.

I have here the contract saying 1000 salary, but the one there [in destination] saying 700, that contract paper is not with me. Now I have 1000 here and according to contract if they check in the bank it will show 1000-1100 [including overtime]. They [employer] have the details of my overtime work and they can also just not show that. So, trying to complain is just a waste of time.

They never gave salary with details. They just gave my salary as one amount. Sometimes I would get a slip saying 1000 and sometimes 1100. I also did not think about the details of my overtime and that slip. I used to just get the money and throw away that slip as I was not thinking of complaining. (Sagar, age-group 25–34)

Losing money, being cheated at some point during migration was extremely common. That, combined with participants' lack of faith in the justice system meant few considered filing official complaints.

Labour migration is an opportunity for men to provide for their families in the way they want. Several men spoke about their growing responsibilities following marriage and having their own family. Not being able to watch their children grow up created sadness for some but this was balanced by knowing had they stayed in Nepal the family may well be living in poverty. However, being away from home for an extensive part of their working life was a huge source of stress for some, which was a sacrifice they had to accept:

We do talk every week. I miss them, they also miss me. But this is how life is. I had to leave my job [in Nepal] and look after my children, my family. I had to close my business... I feel happy thinking about my family but personally I am not happy. (Nabin, age-group 35–44)

Many men also spoke of luck, fate and God as having already predestined their migration outcome and did not consider their own agency and ability to affect change. One participant who worked as a security guard in Afghanistan and experienced an explosion while on duty had the option to return and stay in Nepal. He chose to return to Afghanistan after recovering in Nepal. Despite the dangers, he felt that was the nature of his work and the outcome of any such dangers were in God’s hands.

It was already decided that we all [the Nepali guards who were present during the explosion] would go back to Afghanistan. This is life. Even in my army life we have face many wars and dangers. It all depends on God’s will. If I am unlucky then I will die and if not then I won’t die. (Surya, age-group ≥45)

The idea that migration outcomes were out of their hands also contributed to how men would plan future migrations. Very few described any specific precautions to avoid the problems previously encountered. Instead, they spoke of trusting and having faith that all will be well.

### Support-seeking

Support-seeking strategies were used to cope with ill-health and difficult working conditions. Men sought support from family and friends, recruitment agencies, the embassy, and civil society organisations (CSOs) for emotional support or practical assistance such as obtaining travel documents, filing complaints, seeking employment or financial support.

Several participants contacted friends in the destination for assistance with contract discrepancies when they discovered that these were different to the agreement signed in Nepal:

…we called them [friends in destination] but they said they couldn’t help us. They were also working in a company themselves so they couldn’t help and said it was better for us to just continue to work there. (Ashok, age-group <25)

As mentioned earlier, men who wanted to terminate their contracts early were often told to pay large fines to their employer. Men described seeking financial support from family and friends to manage these situations:

I wanted to come back but was not allowed. The company said I have to buy my own ticket and pay around 70,000 NPR to them as a fine. I needed 2-3 months to earn 70-80,000. So finally, I got financial help from my family to pay the company and returned back to Nepal. (Gopal, age-group 35–44)

One participant reported running away from his employer and seeking refuge at the embassy for assistance. While others described having to go from one group to another without ever receiving assistance, as described below.

…when I said I wanted to come back they [employer] didn’t let me. They didn’t give me any ID so I was not able to come back. After that I went to the embassy… He [person at the embassy] just said go to another company and work. And then I went to another [migrant] organisation … they said we can help you change to another company but cannot help you to go home. No one was helpful. (Dipak, age-group 25–34)

Such unsuccessful attempts when reaching out to official support were commonly described among men that sought help, and these attempts were often the last resort after trying to manage on their own. However, on rare occasions, the authorities were helpful in resolving the issue:

When we didn’t receive salary for 3-4 months, the store [providing groceries on credit] refused to give us anymore foodstuff so then we complained to the police and within 20 days we received 2 months’ salary. (Dhan, age-group ≥45)

This initial success turned out to be a temporary relief as the employer again stopped paying a few months later.

Other participants found that their coping strategy used to address one challenge sometimes introduced new stressors. For example, one participant reported seeking-support from the embassy to file a case against the employer. In doing so, he lost his job and had to rely on friends to find casual labour to support himself.

We asked our friends and brothers if they know about any work. We had no other work; we told them we were unemployed. We did all kinds of work which was very exhausting. We broke many shoes, 14 shoes, in the process. We asked so many people. We used to do whatever work we could get. (Bikash, age-group 25–34)

### Social isolation

Social isolation strategies are based on withdrawing and preventing others from knowing about the stressful situation. Men used these strategies to cope with problems relating to employment conditions and injuries as they felt that sharing with others would not change the situation and may cause more distress. For example, some participants feared disclosing their problems to others may get back to their employer which may worsen the situation, such as their employers refusing to pay them. Others did not want to share their problems especially with their family in Nepal as there was nothing they could do to help. For some men, social isolation strategies were only used while they were in destination, and once back in Nepal, they felt able to share their experiences.

In contrast, some participants reported that even on their return, they continued to conceal their negative experiences from family, friends and acquaintances. Although one suggested that if he were asked directly for advice, he would advise strongly against going to Malaysia. Another participant whose family had not wanted him to migrate in the first place decided not to return to his village at all and was, instead, arranging to re-migrate elsewhere to avoid having to explain to his family why he was back earlier than expected.

Sometimes, the decision to share the problems men were facing with their family in Nepal was not one participants had control over. For example, one participant who had suffered a work-related injury that resulted in him being off work for several months did not inform his family. However, he was eventually forced to tell them three months later, when they asked about remittances.

### Negotiation

Negotiation strategies aims to reach a compromise while protecting one’s own interests. Many men reported using these with supervisors to cope with contract discrepancies and poor living conditions. For example, one explained his situation in Nepal to try to convince his supervisor to pay him his overtime wages which had been underpaid by half for several months.

I worked from morning 8AM to 9PM and got only 2 hours overtime pay. Later I spoke with my supervisor and explained that I have come from very far just to work and earn money so if you don’t pay me the full amount it’s better to send me back. After that I began to receive my 4 hours’ overtime pay. (Dinesh, age-group 35–44)

Going on strike was a common strategy used either individually or collectively with other workers. It was a way to force management to negotiate. Employers that hired workers through a supply company were rarely fully aware of workers’ wage issues, as they pay the supply companies directly, who in turn pay workers. But going on strike even in those situations forces the issue onto the employer who would be dissatisfied with the supply company.

That [to go on strike] was decided by the Nepalis ourselves. It was because we worked in the company, we were not related to the company, just work there. If we don’t get money on time, who could we go with our problems? So, we have a strike in the company, then the people of the company would come and ask us what happened. We then tell them what the issue is. (Karan, age-group <25)

In Karan’s case, their strike action resulted in their employer contacted the supply company who was then forced to dialogue with the workers and manage the situation.

As described earlier, many participants only realised too late that the recruitment agency in Nepal had deceived them. On their return to Nepal, some confronted the agency and attempted to claim some of their money back. Even at that point it was common for some sort of negotiation to take place.

I took some of my friends with me and went there [to the agency]. Then they said “give us 11 days and then we will pay you some money back but on the condition that we will not pay you the cost of one ticket which is 40,000 NPR”. (Rajesh, age-group 35–44)

### Accommodation

Accommodation strategies restructure one’s desired outcome based on the available options, and may be achieved through changing one’s emotional reactions or distracting oneself. They were most commonly used to cope with recruitment issues in the pre-migration phase. Some participants initially had specific destinations or job preferences in mind but these had to be changed due to the delays of obtaining the required documentations, insufficient demand for the specific employment or in their preferred destination. In these cases, participants adjusted their preferences to expediate the process.

Even for participants who experienced contract discrepancy and had unsuccessfully tried to return home later restructured their thinking to convince themselves that staying longer was the right decision. Multiple men described similar stories where having been through the toughest initial period, extending their stay meant they would be able to return home in a better financial position, as explained by one participant:

… it had already been eight months, I had to bring some return after going there. If I could have returned at beginning then it was ok but I couldn’t then because I had to pay money to the company. Now eight months have passed, nine months have passed, and in the ninth month my father told me the loan has been repaid. So, staying five or six more months was not that difficult for me. (Sagar, age-group 25–34)

In addition, accommodation strategies were also used to justify their limited options. For example, losing wages in order to return to Nepal was a better alternative than staying.

### Opposition

Opposition strategies involve directly confronting stressors through retaliating, venting or blaming others. Participants used these primarily to cope with legal issues. For most men, their employers were responsible to obtain and maintain their legal status. Migrants can be left in a highly precarious situation when employers do not fulfil that obligation. One participant who was advised to enter on a tourist visa with the expectation that it will be converted to a work visa, tried initially to negotiate with his employer to apply for the right visa but later resorted to using threats.

I actually worked in his laundrette for two months. The manager had a laundrette, car wash company and hotel as well. And for two months I didn’t get salary and my visa was also about to expire. I had discussion with him but he didn’t care and so I told him I will go to the police and then he was scared because his laundrette and car wash licenses were not renewed. So, then he gave me return ticket. (Rajesh, age-group 35–44)

Once back in Nepal, some participants were no longer fearful of repercussions and confronted the recruitment agency that although these rarely achieved anything more than venting.

I fought with the recruitment agency the day I came back. I shouted at them saying you told me one thing and send me there and you didn’t do what you said you would do. They didn’t have anything to say and then they could send me to a better place, they will do this and that. (Sagar, age-group 25–34)

## Discussion

### Summary of key coping responses

This paper sought to examine the coping strategies male Nepali labour migrants used to manage the range of migration challenges they encountered before, during and after their migration. The main coping strategies Nepali men used to manage the range of stressors they experienced are summarised in Table 3. Overall problem-solving strategies were the most common. This included purchasing safety equipment for themselves, extending their stay, adjusting remittances, returning to Nepal or making up excuses to achieve the desired results, which may be to leave the employment, to reduce exposure to occupational hazards, or to manage on the low wages. Helplessness, support-seeking, social isolation and negotiation strategies were also routinely used depending on the issue confronted. Some men reached out to others for support when they encountered problems; while others preferred to keep their problems to themselves. Negotiation strategies were most often used with employers or the recruitment agency, in an attempt to improve their working situation or to recover some of the fees paid. In some situations, helplessness and accommodation strategies were the only available options after earlier attempts using other strategies. Men then accepted the situation by shifting their perspectives. A minority of men used opposition strategies by threatening the source of the stressors.

**Table 3:** Main coping strategies used by participants by coping families

Problem-solving	Helplessness	Support-seeking	Social isolation	Negotiation	Accommodation	Opposition
seek work abroad	give up control	report to authorities	withdraw from others	talk to supervisor	convince themselves to stay longer	threaten employer
purchase own safety equipment	no attempt to improve situation	call friends, family	do not share actual situation	talk to recruitment agency for refund	adjust destination or work preference	confront agency / agents
reduce expenses remit less often repay loan	rely on luck, fate, destiny	seek work or financial assistance	keep to self	go on strike		
extend stay						
make up excuses						
form groups to use shared facilities						

These findings are important because of the relative dearth of literature on male migrants’ agency, resourcefulness and coping compared to the much larger body of work documenting migrants’ abusive or exploitative circumstances. It was not surprising to learn that when away from home in unfamiliar and often-stress-filled circumstances, men often sought ways to address versus succumb to their problems—or utilise problem-solving strategies and support-seeking—to mitigate any harm that might come to themselves or their family.

A systematic review that assessed coping strategies between male and female migrants found that stressors affected migrants’ earnings were likely to trigger a coping response since the purpose of their migration was economic [33]. Datta et al’s examination of London-based migrant workers found that a wide-range of problems-solving strategies were used to manage situations where their qualifications are not recognised in the UK by still working in the same industry to maintain their skills, even if they were then overqualified and underpaid for the post, or take on additional jobs to increase their income [34]. Additionally, many examples from this and other studies have documented how migrants reduce their living costs in order to increase the amount they can save or remit [35] These ranged from using lower-cost healthcare instead of a clinic [35]; moving to another geographic area [36]; leaving their employment to seek alternative work, even if this means losing their legal status [37–39]. In the first instance, migrants will try to resolve the situation by themselves.

Some studies have found that female migrants more commonly used support-seeking strategies compared to males although it was suggested that the strategies used depended more on the type of stressors encountered [33]. In our study, however, men did reach out to a wide range of individuals and organisations, in both Nepal and the destination country when they encountered difficulties they could not solve by themselves. In line with other studies, support-seeking was commonly reported to deal with practical assistance with employment [34, 40, 41] or financial assistance [34, 36, 42] but also to access healthcare [43] and for emotional support [44]. Men’s willingness to seek assistance suggests that fostering migrant workers’ access to authorities or local organisations can be an important way of supporting workers when they encounter problems, including disputes with employers. Further, migrants who want to leave their employers could be assisted with retrieving or replacing travel documents if these were confiscated by the employer. Participants in this study reported getting the ‘run-around’ from assistance groups, never managing to find the right place to get the help they needed. There were also confusions as to what documents are needed to approach organisations for assistance in the first place. These suggest that embassies, CSOs and others that aim to support migrant workers need to strengthen their capacity to assist migrants through clarifying with their own staff on what assistance are available, by whom, and how these could be accessed. These could reduce stress and confusion for migrants and increase the likelihood they would seek assistance. Ensuring migrants have accurate and up to date information and understand how to access support could improve migrants’ conditions in the destination country.

Importantly, participants reported that they also attempted negotiation strategies, either individually or collectively with other workers, even though most worked in low-waged labour which is typically associated with large power inequalities. This is consistent with studies among garment factory workers in Malaysia [38], domestic workers in Singapore [45], and migrants from new European Union (EU) member states with limited legal and residential rights in the wider EU [46], all of whom negotiated to improve their wages and living conditions. Indeed, participants realised that if they stopped working, the company would suffer financially and this was used to negotiate improvements to their situation. This group of strategies was useful even when the they were not employed directly but through a supply company as demonstrated in this study. This has implications for programs supporting labour organising as it demonstrates that investing in worker-led initiatives will be taken up and utilised by workers to improve their working terms and conditions.

Nearly all the men interviewed were in regular communication with their families in Nepal, which helped them cope with being away from home, and made them feel positive about the sacrifices they made for their family. At the same time, such conversations may have been rather superficial as several did not mention their actual situation. Men were therefore unlikely to have received the support they needed to help them cope with some of the most distressing life events; they had to manage alone, which may have contributed to poor emotional and mental health. Many studies have noted poor mental health among labour migrants, specifically high psychological distress [47, 48]. Additionally, financial issues are likely to mediate poor mental health [49]. According to the GoN report, one of the main causes of death of Nepali migrants is suicide [10]. Further, the reluctance of migrants to share the negative aspects of their experiences may also contribute to the perception that labour migration is always financially rewarding, as those in the community would only see the tangible improvements. Indeed, in some settings labour migration is the only option considered by young men and their families. The perceptions of improved earning potentials and decent living conditions abroad were almost solely based on information from returnees or recruitment agents [12]. Whereas problems encountered are not shared to the same extent, even among friends, meaning aspiring migrants may have a distorted view of labour migration and may be more willing to take riskier decisions, such as taking out larger loans, with the expectation that their migration will lead to financial success [50], and they may be ill-prepared to cope with adversities, including hardships and deceptions.

Coping strategies were sometimes used only after experiencing multiple stressors or when the stressors affected their income, as mentioned previously. Problems with income have direct knock-on effects on their ability to repay loans taken to fund their migration. Loans, the lack of documentation and the desire to prevent their family from distress also shaped the participants’ coping responses. For example, the fact that men did not have copies of both the contract they signed in Nepal and the one signed in destination meant that they had no proof that contract substitutions had taken place, and did not believe they would receive compensation or assistance. Sometimes a range of coping families were used in succession, but when these were unsuccessful in achieving the desired outcome participants resorted to helplessness or social isolation.

Ultimately, when migrants cannot find the supportive assistance for serious employment issues, many consider changing jobs but can be prohibited from doing so because few had access to their passports and in some locations, it is not possible to change employers without the permission or approval from the original employer, or risk losing their legal status in the destination country. These restrictions are common and have been documented among low-skilled migrants in the Middle East, Europe and North America, in a range of temporary guestworker visa programmes [34, 36–38, 40, 51, 52]. These legal restrictions also mean that those who manage to leave and seek work elsewhere can expose them to legal problems, including being arrested or deported or non-payment of wages because employers know they are unlikely to be reported.

The relationship between recruitment agencies, agents, and aspiring migrants is complex, and has been well-documented in studies in both origin and destination countries [53–57]. Agents and agencies play an important role in the migration chain through provision of information and management of the application process, which are often cumbersome and difficult to navigate alone, particularly for those planning their first migration. However, they are also the source of much of migrants’ challenges. Further, some recruitment agencies advise employers to confiscate workers’ passports, salaries, or not allow days off to prevent runaways [38]. The GoN maintains a list of blacklisted agencies that can be consulted but given that many returnee migrants do not file complaints against the agencies it is unclear how accurate the scheme is. A well-publicised rating system that involves more active feedback collected from migrants could be explored. However, most aspiring migrants seek work through advertisements or word of mouth and may not know whether an agency is licensed.

There has been a call for countries to adopt a ‘core rights approach’ to protect labour migrants. Some of these rights could include keeping one’s own identity documents, having equal access to healthcare and legal systems for protection and equal employment conditions such as being permitted to change employers, employment or visa categories and not revoking resident permits in the case of unemployment, for example, due to company closures. In fact, the GoN has bilateral agreements with key destination countries. Some of these agreements include important measures to reduce the burden on migrants through, for example, making employers responsible for recruitment expenses including round trip airfares, stipulating specific dates when salary should be paid, the payment method and granting workers leave in an emergency [58]. This may go a long way to protect Nepali migrant workers. However, past analysis of such agreements has shown that enforcement has been problematic [53, 54, 57, 59].

### Limitations

The original intention for this study was to conduct multiple interviews with the same participant with a view to share the interpretation of the findings from the first interview and to draw out themes for further exploration or explanation. However, as men typically only stayed in Kathmandu for one night, mainly to facilitate the onward journey back to their village, it was not feasible to see the participant for more than once.

This study uses Skinner and Zimmer-Gembeck’s typology of coping families to categorise qualitative narratives. To do so, each stressor and coping response were organised in pairs to indicate which stressor was responded to by which strategy. This method may mask the complex patterns of how stressors and coping interact and affect each other. Nevertheless, given the different ways in which coping strategies have been categorised, using a system that narrows these down to 12 overall typologies based on extensive review and assessment provides an empirically-based approach to organise coping responses and makes findings more comparable to other studies.

Research among mobile populations has challenges due to the difficulties in reaching them. In our earlier study with migrants to assess experiences of exploitation we were only able to locate 40% of those identified six-months earlier [6]. Additionally, migrants that returned to Nepal and agreed to take part in research are likely to be different from those who did not, which may be due to negative or positive experiences. For example, negative experiences preventing migrants from returning may include incarceration, restrictions to their mobility, severe injuries or fatalities. As many migrants intend to re-migrate, some feared that speaking about their experiences may limit their future options for migration. In contrast, those who have been able to earn a decent living abroad are likely to extend their stay. To overcome these challenges, the sampling strategy aimed to recruit a range of participants through key geographic locations in Kathmandu supplemented with referrals from CSO’s. The fieldwork took place ten months after the 2015 earthquake and during the unofficial blockade between Nepal and India, which caused many disruptions to daily life. The interviews were conducted in English with an interpreter. Interviewing as a foreigner may also have brought advantages such as the ability to ask questions that may be considered obvious to locals, particularly on a highly prevalent activity such as labour migration. As highlighted in the findings, some were reluctant to share their experiences with their family and friends. Therefore, having an opportunity to share their stories was a good outlet for some and many described being happy that a foreigner was interested in their experiences and views.

## Conclusion

Labour migration among Nepali males is a highly prevalent livelihood strategy often repeated throughout their working life. This study explored men’s coping responses to a range of stressors encountered during their migration experiences. Problem-solving, support-seeking and helplessness and negotiation were all commonly used strategies. Coping strategies did not always achieve the desired outcome and sometimes introduced new stressors. Those who sought assistance with authorities or CSOs did not always receive help. Improvements to the support mechanisms migrants can access may enable responses as well as enabling worker-led initiatives may lead to improved migration outcomes. As labour migration from Nepal is likely to continue, government and CSOs need to ensure migrants have the support they need to cope with the challenges they may encountere along the way.

## Supplementary Information

**Additional file 1.** The topic guide that was developed for this study tis included in the supplementary file.

## Data Availability

The datasets generated during and/or analysed during the current study are not publicly available due to confidentiality and anonymity issues. The consent procedures did not include data sharing.

## Abbreviations

CSO: Civil society organisations
EU: European Union
GoN: Government of Nepal
UAE: United Arab Emirates
