# Supplementary material for: Coping with migration-related stressors - a qualitative study of Nepali male labour migrants
Source: BMC Public Health. 2021 Jun 12;21:1131. doi: 10.1186/s12889-021-11192-y (PMC8199809; doi:10.1186/s12889-021-11192-y)
Supplement: Supplementary file 1 [file 12889_2021_11192_MOESM1_ESM.docx]

LONDON SCHOOL of HYGIENE & TROPICAL MEDICINE

Could you tell me a bit about your childhood?

- Family, home environment, village life
- School life
- Responsibilities at and outside of home
- Changes in home situation during childhood

Could you talk about your life after school [or in early adulthood]?

- Leaving school – reasons, plans
- Responsibilities
- Relationships
  - work
  - family, marriage – decision, children
- Friends/peers

How did you come to thinking about going for foreign employment?

- Options
- Life outside your village
- influence
- Awareness
  - Others' stories and experiences
- What sort of work did you wanted to do abroad? Thought about having to do other work?
- Hopes? Fears?
- Reactions of family / friends – need convincing?

How did you go about planning your foreign employment?

- Recruitment
- Preparations
- Contacts
- Process

Can you talk about your experience while working abroad?

- Initial impressions
- Day to day living and working conditions
- Relationships at work? Living space? Home (parents/wife/children)?
- Culture, social life, relaxation
- Finances
- Health
- Hopes, concerns, fears
- Preparation

What led you to return to Nepal?

- Unresolved issues at destination
- Perceptions – family, friends, community
- Plans

How would you summarize your experience with foreign employment?

- Positive things, benefits
- Negative things, losses

What do you think about migrant organizations and the government could do for migrant workers?

If you met someone who wanted to migrate, what advice would you give them?
